# Preventive Effect of Platelet-Rich Plasma on Fracture Healing in a Rat Tibial Nonunion Model: A Controlled Laboratory Experiment

**DOI:** 10.7759/cureus.97823

**Published:** 2025-11-26

**Authors:** Tokito Tatsuo, Takayuki Okumo, Itaru Kachi, Yuta Iida, Takumi Nishio, Hiroki Okada, Misako Takizawa, Koji Kanzaki, Yoshifumi Kudo, Masataka Sunagawa

**Affiliations:** 1 Department of Physiology, Showa Medical University Graduate School of Medicine, Tokyo, JPN; 2 Department of Orthopedic Surgery, Showa Medical University Fujigaoka Hospital, Yokohama, JPN; 3 Department of Orthopedic Surgery, Showa Medical University Graduate School of Medicine, Tokyo, JPN

**Keywords:** enhanced fracture healing, histological evaluation, nonunion of tibia, platelet-rich plasma (prp), rat model

## Abstract

Introduction

Nonunion, defined as the absence of progressive healing for more than six months after fracture, remains a major challenge in orthopedic practice, leading to chronic pain, impaired mobility, and reduced quality of life. Platelet-rich plasma (PRP), an autologous concentrate of platelets and growth factors, has been proposed as a biological adjunct for enhancing tissue regeneration. Although PRP has been demonstrated as beneficial for acute fracture healing, its efficacy in established nonunion is unclear.

Methods

We established a rat tibial nonunion model by transverse osteotomy with circumferential periosteal removal and intramedullary fixation. Sixteen male Sprague-Dawley rats (10 weeks old) were randomly assigned to four groups (n = 4/group): Sham (osteotomy only), Nu (nonunion), Nu + PRP, and Nu + Vehicle (saline). Allogeneic pooled PRP was prepared by single centrifugation (200 × g, 7 min) for local injection (50 µL) immediately after surgery and at postoperative weeks 2 and 4. Motor function was assessed using the rotarod test, and bone healing was evaluated by micro-computed tomography (µCT) and histology (Emery fracture healing score) at postoperative week 6.

Results

Compared with whole blood, platelets were enriched approximately 2.5-fold in PRP (*p* = 0.002), with minimal erythrocyte contamination and moderate leukocyte enrichment. There were no significant intergroup differences in rotarod performance at any time point, although the Nu + PRP group consistently trended toward higher fall latencies. µCT revealed significantly greater cortical bridging in the Nu + PRP group compared with the Nu group (*p* = 0.005) and Nu + Vehicle group (*p* = 0.015), with scores comparable to the Sham group. Histological analysis demonstrated mature trabecular bone and a higher Emery fracture healing score in the Nu + PRP group relative to the Nu group (*p* = 0.005) and the Nu + Vehicle group (*p* = 0.015), approaching those of the Sham group.

Conclusions

Radiological and histological analyses in a validated rat tibial nonunion model revealed that repeated PRP administration significantly improved bone repair. Although functional recovery was not significantly improved within six weeks, these findings support PRP as a biologically active and clinically promising adjunct for challenging cases of fracture nonunion.

## Introduction

Fracture healing is a complex, tightly regulated biological process involving inflammation, cell proliferation, angiogenesis, and bone remodeling [1]. Under normal circumstances, this cascade results in successful union; however, approximately 5%-10% of fractures fail to consolidate within the expected period, progressing to delayed union or nonunion [2]. Delayed union describes a situation in which fracture healing is slower than expected but still progressing, whereas nonunion reflects a cessation of progressive healing, typically after more than six months [3]. Despite advances in surgical and biological techniques, fracture nonunion remains a significant challenge in orthopedic practice, resulting in prolonged disability, diminished quality of life, and substantial socioeconomic impact due to extended treatment duration, loss of productivity, and increased healthcare expenditure [4].

The pathogenesis of nonunion is multifactorial, encompassing mechanical instability, compromised vascularity, infection, systemic comorbidities (e.g., diabetes mellitus or osteoporosis), smoking, and adverse effects of medications, such as nonsteroidal anti-inflammatory drugs and corticosteroids [5]. At the tissue level, nonunion is characterized by disruption of the normal fracture healing cascade, with persistent inflammation, impaired angiogenesis, insufficient recruitment of progenitor cells, and defective osteoblast differentiation [6].

Current therapeutic approaches aim to restore both mechanical stability and biological activity. Standard interventions include surgical revision with internal or external fixation, bone grafting (autograft or allograft), and the use of bone morphogenetic proteins (BMPs) [7]. Although these methods achieve union in many cases, each has notable drawbacks: autografts have limited availability and cause donor-site morbidity [8], allografts carry risks of immunogenicity and disease transmission [9], and BMPs are costly and may cause adverse effects, such as ectopic ossification [10]. Noninvasive adjunctive therapies, including low-intensity pulsed ultrasound (LIPUS) therapy, have demonstrated variable success, particularly in cases of established nonunion [11].

Biological therapies have attracted considerable interest in recent years as potential adjuncts to enhance fracture healing [12]. Among them, platelet-rich plasma (PRP) has emerged as a promising option because of its autologous origin, ease of preparation, and high concentration of bioactive molecules [13]. Upon activation, PRP releases a cocktail of growth factors, including platelet-derived growth factor (PDGF), transforming growth factor beta (TGF-β), and vascular endothelial growth factor (VEGF), which synergistically promote angiogenesis, recruit mesenchymal stem cells, stimulate osteoblast proliferation, and facilitate extracellular matrix synthesis [14]. Beyond hemostasis, platelets themselves orchestrate inflammation, angiogenesis, and matrix remodeling, providing the biological rationale for PRP use [15].

Despite encouraging in vitro and in vivo data, clinical outcomes for PRP in fracture healing are inconsistent [16,17], which may be due to differences in preparation techniques, platelet concentration, leukocyte content, and activation protocols [18]. Furthermore, most preclinical studies have focused on acute fracture models [19], whereas the therapeutic potential of PRP in biologically compromised environments, such as nonunion, remains underexplored.

Evaluation of the efficacy of PRP in biologically compromised fracture healing requires a reproducible and clinically relevant nonunion model [20]. The tibia, particularly the distal third, is especially vulnerable to nonunion, partly because of its subcutaneous location, limited soft tissue coverage, and relatively poor blood supply [21]. Therefore, we used a validated rat tibial nonunion model created by circumferential periosteal stripping to mimic high-risk tibial shaft fractures with severe periosteal and soft-tissue injury that are prone to nonunion. In this biologically compromised setting, we investigated whether local administration of PRP could enhance bone repair and prevent progression to nonunion. Using this approach, we aimed to elucidate the potential role of PRP as a biologically based adjunct in the management of impaired fracture healing.

## Materials and methods

Study design and ethical approval

This study was designed as a controlled laboratory experiment using a validated rat tibial nonunion model to evaluate the effect of PRP on bone healing. Group allocation was performed using a computer-generated randomization sequence by an investigator who was not involved in outcome assessment, and all outcome assessors were blinded to group assignment. The sample size (n = 4 per group) was determined a priori based on feasibility considerations and previous studies using similar nonunion models, and no formal power calculation was performed; this is acknowledged as a limitation of the study. The inclusion criteria were male Sprague-Dawley rats that underwent successful intramedullary fixation and periosteal stripping. The exclusion criteria were perioperative death, surgical fixation failure, or postoperative complications preventing evaluation. All procedures were approved by the Animal Care and Use Committee of Showa Medical University (Approval No. 124056) and reported in accordance with institutional policies and the ARRIVE (Animals in Research: Reporting of In Vivo Experiments) guidelines [22].

Animal model and surgical procedures for tibial nonunion

**Figure 1:**
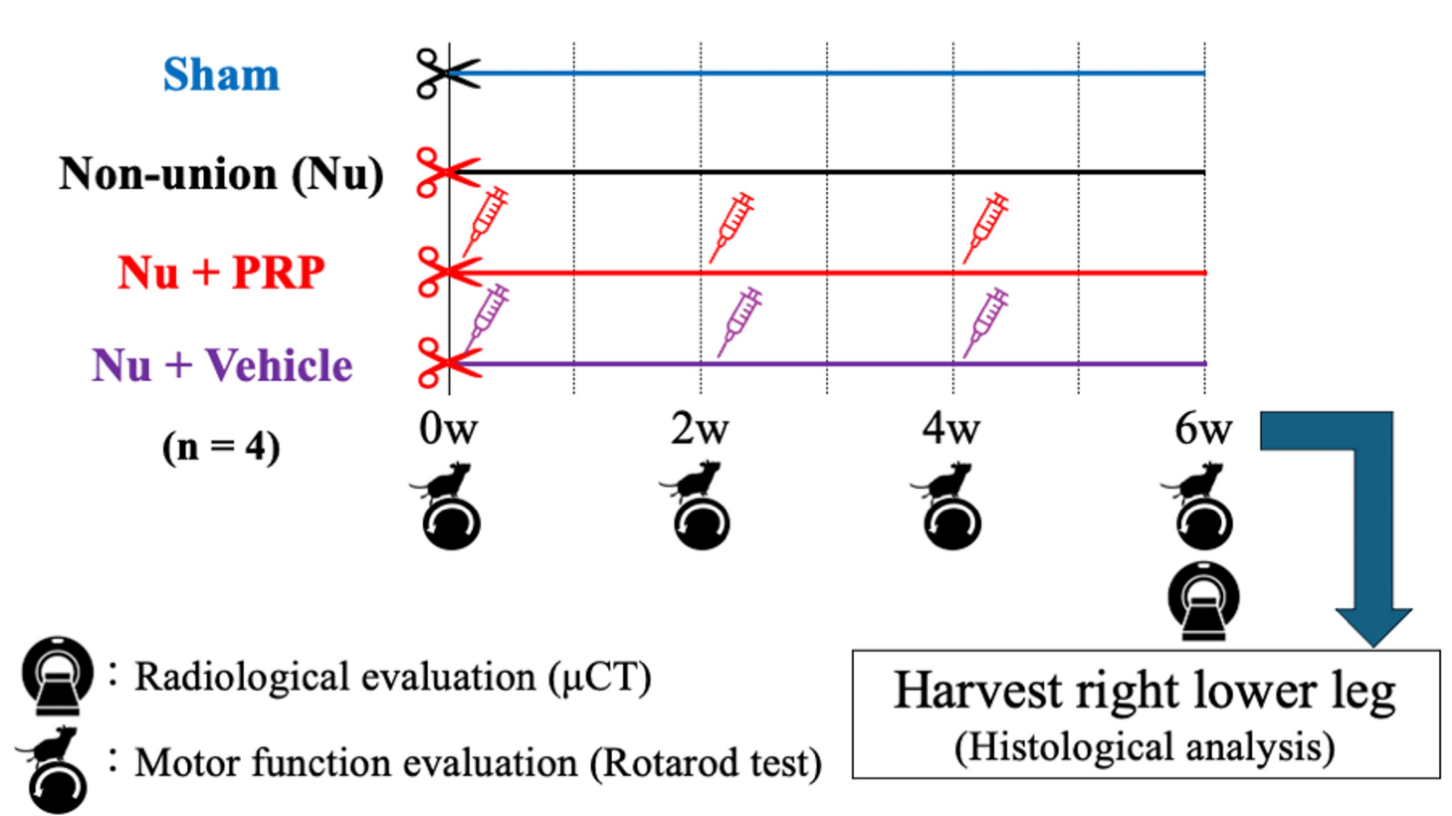
Schematic illustration of the experimental protocol. Sixteen rats were randomly allocated to four groups: Sham, Nonunion (Nu), Nu + PRP, and Nu + Vehicle. After tibial osteotomy, the rats underwent motor function assessment by rotarod test at baseline (week 0) and at postoperative weeks 2, 4, and 6; radiological evaluation by μCT at postoperative week 6; and histological analysis at postoperative week 6 (n = 4/group). Nu: Nonunion, PRP: Platelet-rich plasma, μCT: Micro-computed tomography. Image Credit: Tokito Tatsuo

Sixteen male Sprague-Dawley rats (10 weeks old, body weight: 300-350 g) were randomly assigned to four groups (n = 4/group): Sham, Nu (nonunion), Nu + PRP, and Nu + Vehicle. No animals were excluded or replaced during the study. To avoid potential variability associated with the hormonal effects of the estrous cycle, only male rats were used. The rats were housed in conventional open cages (plastic cages with wire lid tops) in a temperature- and humidity-controlled environment (24 °C-26 °C, humidity: 40%-60%, 12-h light/dark cycle), with access to food and water ad libitum. Cage location was randomized to minimize confounding. Animals were housed in groups of two to three per cage with hardwood bedding, paper nesting material, and cardboard tubes as environmental enrichment. Animals were acclimatized for seven days before surgery. All surgical procedures were performed under aseptic conditions, and the operative field was prepared using povidone-iodine and sterile draping under general anesthesia induced and maintained with 2-3% isoflurane in oxygen delivered via a nose cone. The right tibia was exposed in all groups, and osteotomy was performed at the mid-diaphysis using a micro saw. The Sham group underwent osteotomy with the periosteum preserved, so that a nonunion state was not induced. In the Nu, Nu + PRP, and Nu + Vehicle groups, the periosteum was circumferentially excised around the osteotomy site (approximately 5 mm) to induce nonunion, and 0.8-mm Kirschner wires were inserted for intramedullary fixation. The Nu + PRP group was treated with local injections of PRP, and the Nu + Vehicle group was treated with local injections of equivalent volumes of saline as the vehicle control (Figure 1). At wound closure, the skin and periosteal tissues around the osteotomy site were infiltrated with 0.25% bupivacaine (up to 2 mg/kg) to provide local analgesia. Postoperative systemic analgesia was provided with subcutaneous buprenorphine (0.03-0.05 mg/kg) every 12 h for 48 h. Animals were monitored daily, and humane endpoints were predefined according to institutional ethical standards. 

PRP preparation and administration

Whole blood (8 mL/rat) was collected from five donor Sprague-Dawley rats (10 weeks old) under general anesthesia with isoflurane inhalation and pooled to prepare allogeneic PRP. A single centrifugation protocol was used (200 ×g, 7 min) [23]. After harvesting the 400 µL of PRP with buffy coat, enrichment was confirmed by determining the platelet and leukocyte counts in paired samples of whole blood and PRP. Based on the moderate leukocyte enrichment and inclusion of the buffy coat, this preparation corresponded to leukocyte-rich PRP. PRP was not exogenously activated before injection and was administered in its native liquid form. To preserve its bioactivity, the prepared PRP was stored at −80°C until use. Immediately after surgery and again at postoperative weeks 2 and 4 (under brief anesthesia), each rat in the Nu + PRP group was injected with 50 µL of pooled PRP, and each rat in the Nu + Vehicle group was injected with an equivalent volume of saline at the osteotomy site, whereas each rat in the Nu + Vehicle group was injected with an equivalent volume of saline. All injections were performed under brief isoflurane anesthesia via a nose cone.

Motor function evaluation

The rotarod test was used as a behavioral assay to evaluate motor function and discomfort in the model rats. This test is widely used to assess motor coordination and balance in various neurological and musculoskeletal disease models [24]. Briefly, each rat was placed on a gradually accelerating motorized rotating rod (LE8305, diameter: 60 mm, lane width: 75 mm; Panlab Harvard Apparatus, Barcelona, Spain). The time taken for the rat to lose balance and fall from the rod (fall latency) was recorded, with longer latencies indicating better locomotor performance. The rats were familiarized with the apparatus for 2 consecutive days before the start of the experiment by keeping them on the rod at 4 rpm for 180 s. Testing was conducted at baseline and at postoperative weeks 2, 4, and 6. On each test day, three trials were performed per animal, and the mean fall latency was used for analysis after confirming adequate acclimatization. All behavioral assessments were performed by an investigator who was blinded to group allocation.

Radiological evaluation

At postoperative week 6, micro-computed tomography (µCT) was performed using an R_mCT-2 (tube voltage: 90 kV, current: 160 µA, field of view: 60 mm; Rigaku Corporation, Tokyo, Japan). The acquired DICOM data were reconstructed and evaluated using Miele-LXIV DICOM Workstation and Viewer v9.49 software (developed by Alex Bettarini; macOS version distributed via Mac App Store), and multiplanar reconstruction data were processed using the 3D maximum intensity projection function to generate three-dimensional images. The images were rotated to display frontal and lateral views of the tibia for evaluation, the medial and lateral cortices were assessed in the frontal view, and the anterior and posterior cortices were assessed in the lateral view. The presence of continuous cortical bone was scored as 1 point, and the absence of continuity was scored as 0 points in four planes (medial, lateral, anterior, and posterior). The four scores were summed, yielding a total of 0-4 points for each specimen. All measurements were independently performed by two blinded raters (TT and IK). Intraclass correlation coefficients were used to assess intrarater and interrater reliability (ICC(1,2) and ICC(2,2), respectively).

Histological evaluation

For histological analysis, the rats were deeply anesthetized via intraperitoneal injection of 50 mg/kg sodium pentobarbital (Somnopentyl; Kyoritsu Seiyaku, Tokyo, Japan) and subsequently euthanized by transcardial perfusion with phosphate-buffered saline (pH 7.4), followed by whole-body perfusion fixation with 4% paraformaldehyde. After excision, the tibiae were immersed in 4% paraformaldehyde for three days to ensure complete fixation, followed by decalcification in 20% disodium ethylenediaminetetraacetic acid (EDTA) solution for approximately three weeks. Then, the samples were embedded in paraffin blocks for slicing into 4-μm-thick serial sections (200 μm intervals in both medial and lateral directions from the midline) using a rotary microtome (REM-700; Yamato Kohki Industrial Co., Ltd., Saitama, Japan). The sections were mounted on glass slides, stained with toluidine blue, and examined under light microscopy (BX53; Olympus, Tokyo, Japan). Histological evaluation was performed using the Emery fracture healing score, which reflects the maturity of bone-healing tissue and the progress of fracture healing [25]. Three independent blinded raters (TT, TO, and IK) each performed two independent evaluations per specimen. The two readings were averaged to obtain a rater-specific mean for each rater. The final histological score for each specimen code was calculated as the mean of the three rater-specific means. When multiple sections were obtained from the same animal, the animal-level score was determined as the average of the final scores of all sections from that animal. Intrarater and interrater reliability were assessed using ICC(1,2) and ICC(2,3), respectively.

Statistical analysis

All statistical analyses were performed using JMP Pro v17 software (SAS Institute Inc., Cary, NC, USA). Data were presented as the mean ± standard deviation. Due to the small sample size, the normality of the data distribution for each group was assessed as a reference. Based on this approach, parametric tests were applied to all groups. Comparisons between groups were performed using one-way analysis of variance (ANOVA), followed by Tukey honestly significant difference (HSD) test. A two-sided p-value <0.05 was considered statistically significant.

## Results

Hematological characterization of PRP

**Figure 2:**
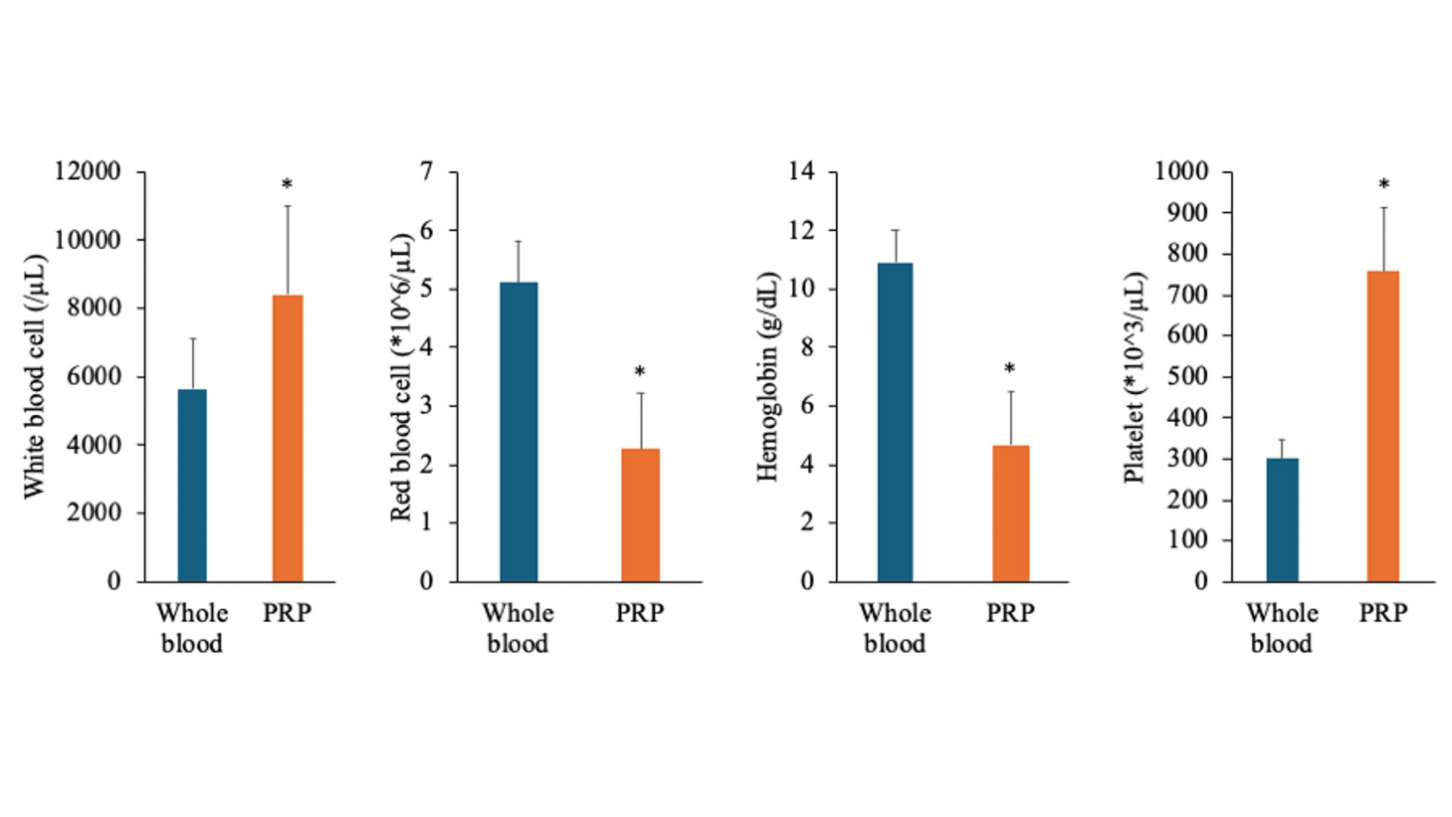
Hematological characterization of platelet-rich plasma. Hematological parameters were compared between whole blood and PRP. Platelet counts were significantly increased in PRP, whereas RBCs and Hb were significantly reduced. WBCs showed a modest increase. Data are presented as mean ± SD (n = 5). ^*^P < 0.05 vs. whole blood based on the paired t-test. Hb: Hemoglobin, PLT: Platelet, PRP: Platelet-rich plasma; RBC: Red blood cell, WBC: White blood cell.

Hematological parameters were compared between whole blood and PRP to assess PRP quality. The platelet concentration in PRP increased approximately 2.52-fold compared with whole blood (301 × 10³/μL vs. 757.60 × 10³/μL, p = 0.002), confirming successful platelet enrichment. Red blood cell counts decreased (5.11 × 10⁶/μL vs. 2.26 × 10⁶/μL, p < 0.001), and hemoglobin decreased (10.92 g/dL vs. 4.68 g/dL, p < 0.001), indicating minimal erythrocyte contamination. White blood cell counts increased (5,646/μL vs. 8,414/μL, *P* = 0.033), suggesting a degree of leukocyte enrichment related to the centrifugation protocol (Figure 2).

Motor function assessment using the rotarod test

**Figure 3:**
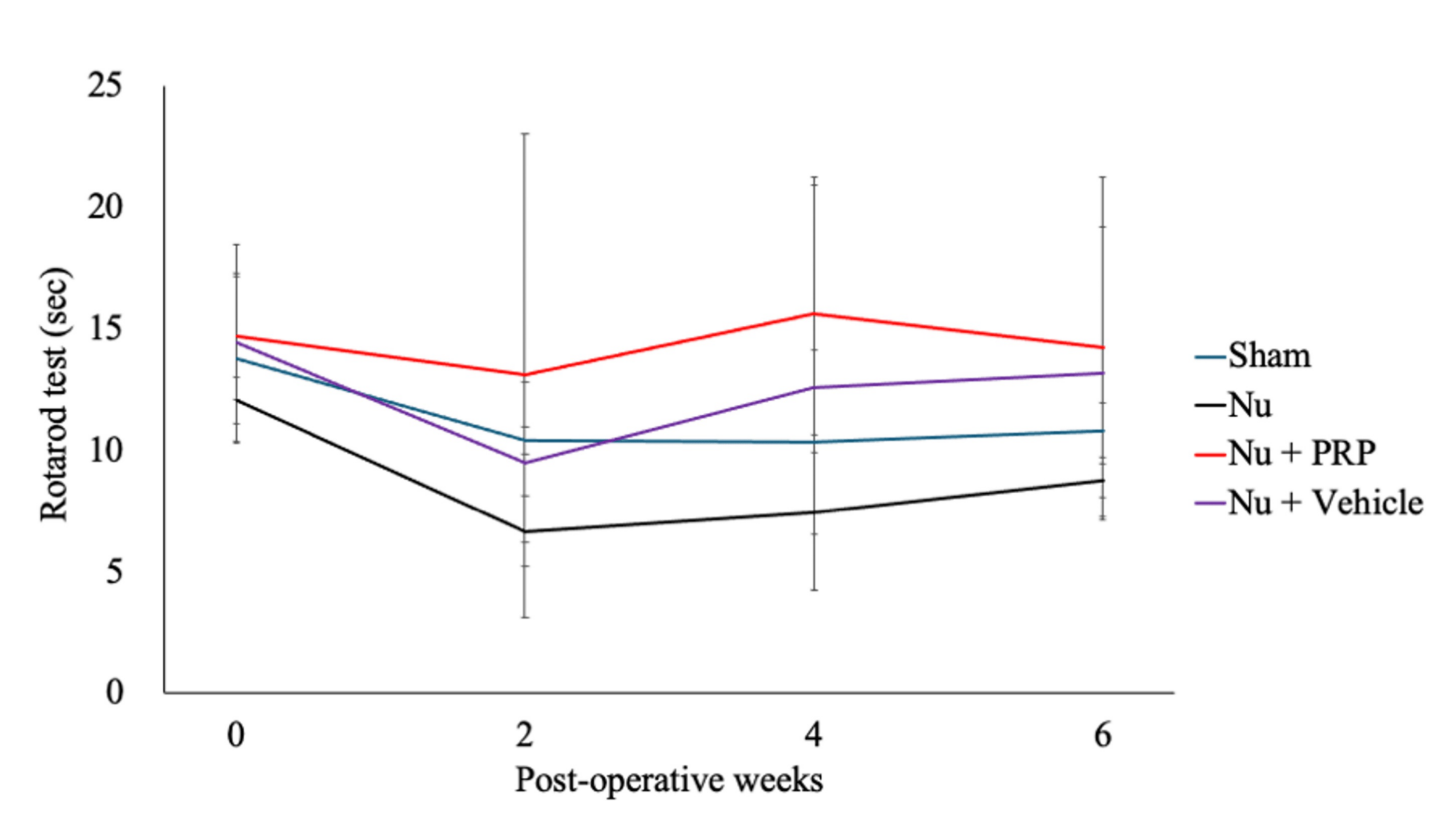
Motor function evaluation using the rotarod test. Fall latency (seconds) was assessed at baseline (week 0) and postoperative weeks 2, 4, and 6 for all groups (Sham, Nu, Nu + PRP, and Nu + Vehicle). Although no statistically significant differences were observed among the groups at any time point, the Nu + PRP group consistently trended toward better motor performance. Nu: Nonunion, PRP: Platelet-rich plasma.

At baseline (week 0), motor function assessment using the rotarod test did not show any significant differences among the groups, confirming comparable preoperative motor function (Sham, 13.75 ± 3.40 s; Nu, 12.08 ± 0.96 s; Nu + PRP, 14.67 ± 2.60 s; Nu + Vehicle, 14.42 ± 4.09 s; *P*-ANOVA = 0.626). By postoperative week 2, the Nu group exhibited a marked reduction in fall latency (6.67 ± 1.44 s), consistent with nonunion-related impairment. In contrast, the Nu + PRP group maintained higher values (13.08 ± 9.98 s), and the Sham and Nu + Vehicle groups showed a fall latency of 10.42 ± 0.57 s and 9.50 ± 3.29 s, respectively (*P*-ANOVA = 0.429). At postoperative week 4, the Nu + PRP group achieved the longest latency (15.58 ± 5.67 s), followed by the Nu + Vehicle (12.58 ± 8.35 s), Sham (10.33 ± 3.78 s), and Nu (7.42 ± 3.20 s) groups (*P*-ANOVA = 0.261). By postoperative week 6, all groups showed some improvement. However, the Nu + PRP group continued to have the longest latency (14.25 ± 6.99 s), followed by the Nu + Vehicle (13.17 ± 6.02 s), Sham (10.83 ± 1.11 s), and Nu (8.75 ± 0.69 s) group. No statistically significant intergroup differences were observed at any time point (week-specific* P*-ANOVA >0.05) (Figure 3).

Radiological evaluation by μCT

**Figure 4:**
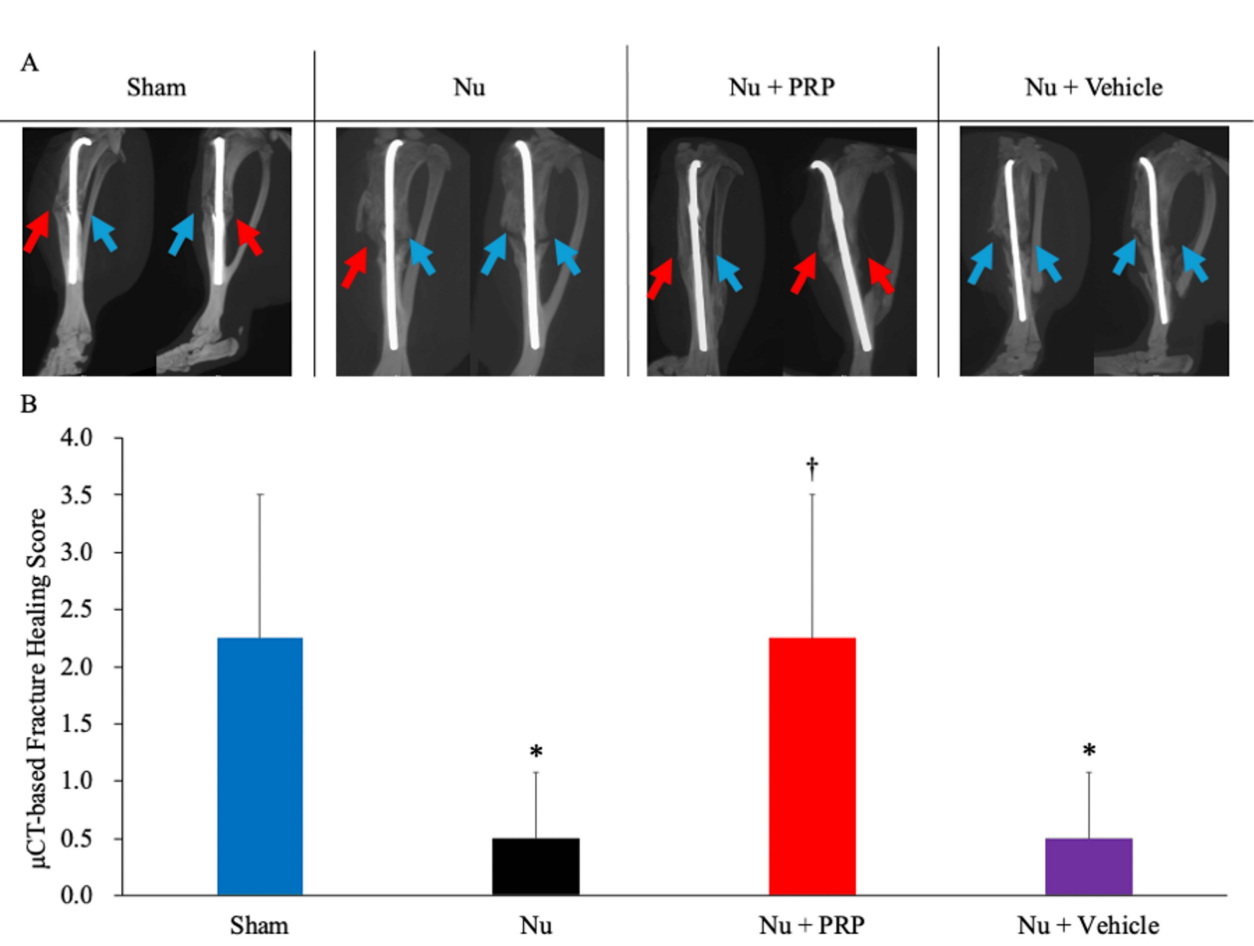
Radiological evaluation of bone healing as assessed using μCT. (A) Representative reconstructed images showing cortical continuity in the Sham and Nu + PRP groups and persistent fracture gaps in the Nu and Nu + Vehicle groups. Blue arrows: persistent fracture gaps; red arrows: cortical bridging. (B) Quantitative fracture healing scores based on cortical bridging in four planes (medial, lateral, anterior, and posterior). Data are presented as mean ± SD. ^*^P < 0.05 vs. the Nu group, ^†^*P* < 0.05 vs. the Nu + Vehicle group based on the Tukey honestly significant difference (HSD) test. μCT: Micro-computed tomography, Nu: Nonunion, PRP: Platelet-rich plasma.

The intrarater reliability (ICC(1,2)) was 0.863, and the interrater reliability (ICC(2,2)) was 0.758 for μCT evaluation. At postoperative week 6, μCT revealed notable differences in bone healing among the groups (Figure 4A). The Sham group showed continuous cortical bridging in multiple planes (mean score, 2.25 ± 1.26; 0-4 scale). The Nu and Nu + Vehicle groups showed persistent fracture gaps with minimal callus formation (each 0.50 ± 0.58), consistent with nonunion. The Nu + PRP group exhibited improved healing, with partial to complete bridging (2.25 ± 1.26), comparable to the Sham group. One-way ANOVA indicated a significant group effect (*P* = 0.029). Tukey HSD test revealed that the Nu + PRP group had significantly higher scores than the Nu group (*P* = 0.005) and the Nu + Vehicle group (*P* = 0.015), while no significant difference was observed between the Nu + PRP and Sham groups (*P* = 0.106) (Figure 4B).

Histological evaluation

**Figure 5:**
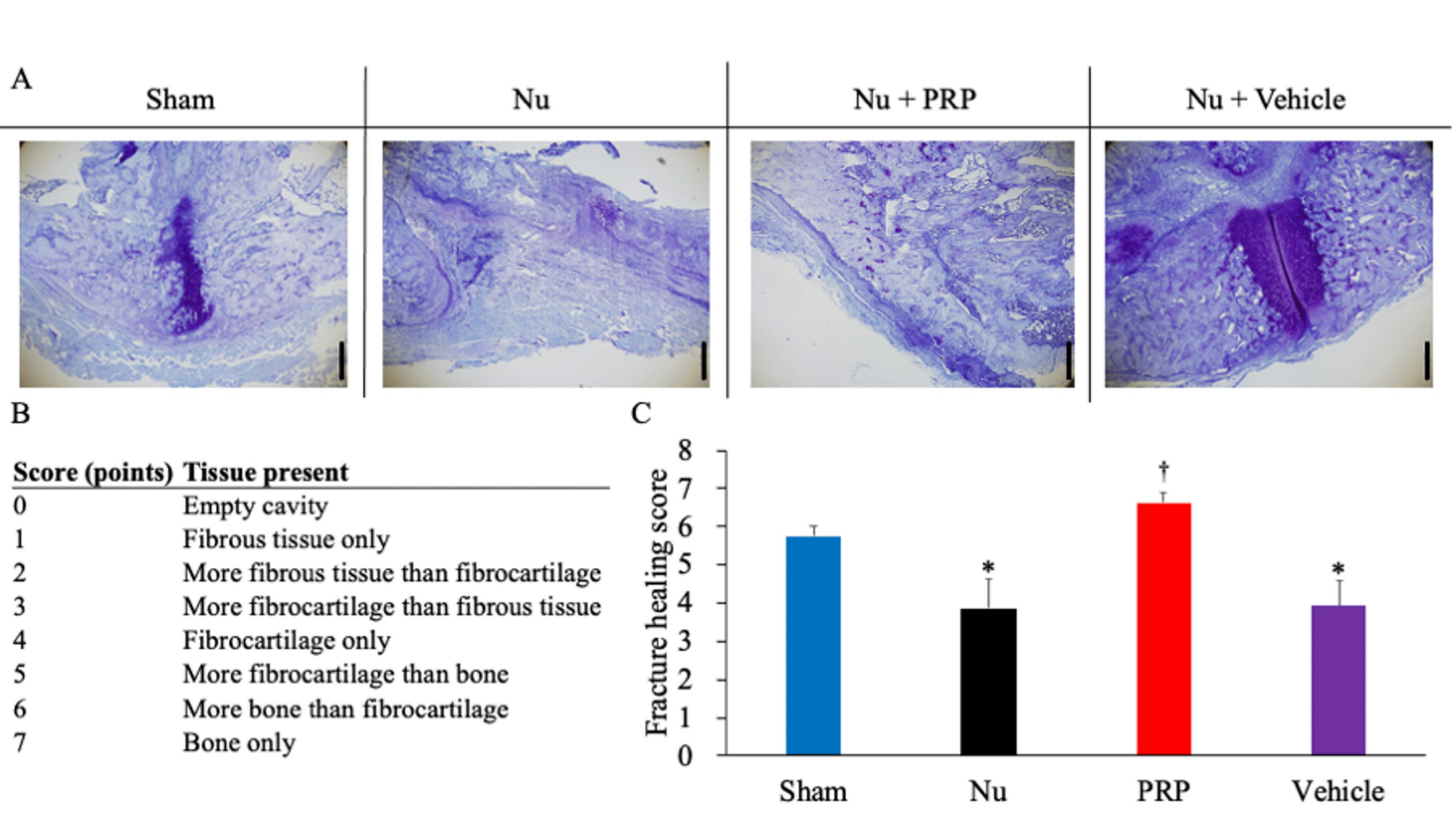
Histological evaluation of fracture healing. (A) Representative toluidine blue-stained sections. The Sham and Nu + PRP groups showed mature trabecular bone and organized marrow architecture. In contrast, the Nu and Nu + Vehicle groups exhibited fibrous tissue and incomplete endochondral ossification. Magnification: ×40. Scale bars = 200 µm. (B) Emery fracture healing scores (0–7). (C) Representative composite summary of histological scores, demonstrating the distribution of Emery fracture healing scores across groups. Data are presented as mean ± SD. ^*^P < 0.05 vs. the Nu group, ^†^*P* < 0.05 vs. the Nu + Vehicle group based on the Tukey significant difference (HSD) test. Nu: Nonunion, PRP: Platelet-rich plasma.

The intrarater reliability (ICC(1,2)) was 0.818, and the interrater reliability (ICC(2,3)) was 0.817 for histological evaluation. Toluidine blue staining demonstrated mature trabecular bone with organized marrow spaces in the Sham group. Advanced healing was observed in the Nu + PRP group, characterized by well-developed trabeculae, active remodeling, and restored marrow spaces. In contrast, the Nu and Nu + Vehicle groups showed persistent fibrous tissue, limited cartilage and bone formation, and incomplete endochondral ossification (Figure 5A). Quantitatively, Emery scores (0-7; Figure 5B) were 5.02 ± 0.90, 2.96 ± 0.84, 5.64 ± 0.70, and 3.36 ± 1.06 in the Sham, Nu, Nu + PRP, and Nu + Vehicle groups, respectively (n = 4/group). ANOVA showed a significant group effect (p = 0.003). Tukey HSD test indicated that the Nu + PRP group scored significantly higher than the Nu and Nu + Vehicle groups (p = 0.005 and p = 0.015, respectively), and the Sham group scored significantly higher than the Nu group (p = 0.028). All other pairwise comparisons were not significant. These results suggest that PRP improves radiological union tendencies and restores histological bone quality toward levels observed under conditions of normal physiological healing, even within a nonunion environment (Figure 5C).

## Discussion

This study demonstrated that repeated local administration of PRP significantly enhanced bone repair in a rat tibial nonunion model. Radiological and histological evaluations consistently showed that PRP-treated model rats exhibited more advanced bone regeneration compared with untreated controls, approaching the healing profile of the Sham group. By generating nonunion through circumferential periosteal stripping, our model mimicked high-risk tibial shaft fractures with severe periosteal and soft-tissue injury in which fracture healing is biologically compromised [19,20]. These findings provide novel preclinical evidence that PRP restores bone healing, even under biologically compromised conditions of nonunion, highlighting PRP as a promising adjunctive therapy in cases where fracture healing is biologically compromised. Moreover, the ICC values indicated substantial inter- and intrarater reliability, demonstrating the robustness of the radiological and histological assessments.

Although our findings support a beneficial effect of PRP, contemporary syntheses of the clinical literature reach nuanced conclusions. A focused meta-analysis on long-bone delayed union and nonunion reported higher healing rates and shorter times to union with PRP compared with controls, while emphasizing the need for larger trials to address functional outcomes and long-term safety [17]. An umbrella meta-analysis that re-pooled individual studies found overall improvements in both healing rate and healing time. However, because only healing time remained significant when individual data were reanalyzed, the overall certainty was rated as requiring confirmation [26]. A comprehensive review of preclinical and clinical studies concluded that although preclinical evidence was consistently favorable, clinical data more reliably showed shortened healing duration rather than a uniformly improved healing rate, while functional outcomes remained inconsistent [27]. In our model, PRP clearly improved structural healing on µCT and histology, whereas rotarod performance did not differ significantly among groups, mirroring the discrepancy between radiographic and functional outcomes seen in some clinical series. Collectively, these converging and diverging findings suggest that PRP most robustly accelerates the biology of repair, whereas its effect on pain and function may depend strongly on patient- and fracture-specific factors, concomitant surgery, and rehabilitation protocols.

From a conceptual standpoint, our work also contributes to theory building around biological adjuncts for nonunion. The diamond concept of fracture healing posits that successful repair depends on the interplay of mechanical stability, osteogenic cells, growth factors, and an appropriate scaffold [7]. By selectively impairing the biological environment through periosteal stripping while maintaining mechanical stability with intramedullary fixation, our model isolates the contribution of biological augmentation with PRP in a nonunion-prone setting. However, the present study interrogated only a single PRP formulation, dosing schedule, and mechanical environment. As such, our ability to refine theory regarding the optimal composition, timing, and mechanical context for PRP use remains limited, and the conceptual model we propose for PRP-mediated rescue of nonunion should be regarded as preliminary.

The therapeutic benefits of PRP are biologically plausible, given its release of multiple bioactive factors upon platelet activation, including PDGF, TGF-β, VEGF, and insulin-like growth factor 1. These factors act synergistically to promote mesenchymal stem cell recruitment and proliferation, angiogenesis, osteoblast differentiation, and extracellular matrix synthesis [6]. In our study, hematological characterization of PRP confirmed an approximately 2.5-fold enrichment of platelets, with minimal erythrocyte contamination and moderate leukocyte content. The latter indicates that the preparation was closer to leukocyte-rich PRP, which may exert both anabolic and immunomodulatory effects, making it potentially advantageous in the chronic inflammation environment of nonunion [28].

In this study, the rotarod test results did not reveal statistically significant functional improvement following PRP treatment. The absence of significant intergroup differences in motor performance may be partly attributable to the intramedullary fixation with Kirschner wires, which could have provided sufficient mechanical stability to minimize functional differences among groups despite histological disparities, suggesting that rotarod performance is influenced by pain-related behavior rather than bone healing itself [24]. On the other hand, μCT confirmed that PRP promoted cortical bridging in multiple planes, which is a key determinant of mechanical stability in experimental fracture healing [29]. Histologically, the PRP-treated group achieved higher Emery fracture healing scores, showing mature trabecular bone formation with organized marrow architecture. In contrast, untreated nonunion groups remained dominated by fibrous tissue and incomplete endochondral ossification.

Several other biologically based strategies have been explored for managing fracture nonunion. BMPs, particularly BMP-2 and BMP-7, are potent osteo-inductive growth factors that drive mesenchymal progenitor cells toward osteoblastic differentiation. However, clinical use is limited due to high cost, restricted availability, and adverse events, such as ectopic ossification, radiculitis, and excessive swelling, particularly with recombinant human BMP-2 [10,30]. Cell-based therapies, such as mesenchymal stem cell transplantation, aim to directly supplement osteoprogenitor cells at the fracture site, and preclinical studies and early clinical trials show promising results. However, clinical application of such cell-based strategies is hindered by technical complexity, regulatory challenges, the need for specialized cell-processing facilities, and uncertainty surrounding cell survival and functional integration [31]. Physical stimulation modalities, including LIPUS, attempt to enhance repair by biophysically stimulating bone tissue. Although a large randomized controlled trial involving acute tibial fractures failed to demonstrate the efficacy of LIPUS, some systematic reviews and clinical reports suggest potential benefits of LIPUS in established nonunion [11]. Thus, the overall effectiveness of LIPUS remains uncertain. Taken together, current adjunctive strategies for fracture healing are promising but remain constrained by cost, complexity, or inconsistent evidence.

Against this backdrop, PRP offers a unique balance of practicality and biological activity. Unlike BMPs, PRP is autologous, and its favorable safety profile enables repeated application without major procedural risks [6,13,15]. Moreover, PRP can be easily integrated into standard surgical workflows without needing specialized facilities, and there are no regulatory hurdles to overcome. Thus, PRP is a clinically attractive adjunct that may complement or provide an alternative to other biological or physical approaches for fracture nonunion. Moreover, the dosing regimen adopted in this study (administration immediately after surgery and again at postoperative weeks 2 and 4) may have provided a sustained biological stimulus, and this strategy may be invaluable in cases of impaired healing with diminished local regenerative activity. Nevertheless, considering the heterogeneity of PRP preparations reported in the literature [6], future studies are necessary to refine dosing intervals and formulations.

This study has several limitations. First, the sample size was small, which limited statistical power, particularly for functional outcomes. Second, we used allogeneic pooled PRP instead of autologous PRP (the clinical standard), which may have influenced immune responses and limits direct clinical translation. Third, radiological assessment relied on cortical-bridging scores without quantitative μCT parameters (e.g., bone volume fraction and tissue mineral density). Fourth, molecular analyses and biomechanical testing were not performed. Finally, although the rat tibial nonunion model recapitulates key features of human pathology, rodent bone biology differs from humans. These methodological constraints also limit the extent to which our findings can be generalized across different nonunion etiologies, mechanical environments, and PRP formulations; consequently, the theoretical implications of this work for clinical decision-making should be interpreted with appropriate caution. Thus, future validation in large-animal models and clinical studies will be essential.

## Conclusions

Repeated local administration of PRP significantly enhanced bone repair in a validated rat tibial nonunion model, as evidenced both radiologically and histologically. With its favorable safety profile and ease of application, PRP may serve as a practical and biologically active adjunct for managing fracture nonunion. Further clinical studies are warranted to optimize dosing schedules, determine the most effective leukocyte composition, and identify patient populations most likely to benefit from this approach.
